# Association between smoking, smoking cessation and serum α-klotho levels among American adults: National Health and Nutrition Examination Survey

**DOI:** 10.1371/journal.pone.0300562

**Published:** 2024-03-18

**Authors:** Ting Liu, Meihua Song, Jie Li, Yumei Zhao, Weiming Zhong

**Affiliations:** 1 Department of Endocrinology, Beijing Liangxiang Hospital, Capital Medical University, Beijing, China; 2 Department of Public Health, Beijing Normal University Hospital, Beijing, China; 3 Department of Gastroenterology, Chinese Medicine Hospital of Longquan, Chengdu, China; 4 The second department of Endocrinology, The first affiliated hospital of Kunming Medical University, Kunming, China; 5 Department of Gastroenterology, The 904 Hospital of the PLA Joint Logistics Support Force, Wuxi, China; University of Palermo, ITALY

## Abstract

α-klotho is an anti-aging protein. The correlation between smoking, smoking cessation and serum α-klotho levels remains controversial. The aim of this study was to investigate the association between smoking, smoking cessation and serum α-klotho levels. This cross-sectional study finally included 4877 participants, aged 40–79 years, who participated in the National Health and Nutrition Examination Survey studies from 2013 to 2016. Of these, 2312 (47.4%) were men and 894 (18.3%) were current smokers, and the mean age of the participants was 57.8±10.7 years. Multivariate linear regression modeling was used to assess the association between smoking, smoking cessation and serum α-klotho levels. After adjustment for multiple confounders, this study observed that smoking was negatively associated with serum α-klotho levels (β: -58.3; 95% confidence interval CI: -82.0 to -34.6; p<0.001), whereas smoking cessation was positively associated with serum α-klotho levels (β: 52.3; 95% CI: 24.1 to 80.6; p<0.001). In subgroup and interaction analyses, *p*-value for the interaction between smoking and race on serum klotho levels was found to be less than 0.001. The correlation between smoking, smoking cessation and serum α-klotho levels remained stable after propensity score matching (β: -54.1; 95% CI: -81.5 to -26.7; *p*<0.001, β: 54.8; 95% CI: 24.2 to 85.4; *p*<0.001). In a large sample population, the present study found that smoking, smoking cessation and serum α-klotho levels were associated in opposite directions.

## Introduction

Smoking is an independent risk factor for cardiovascular disease and the leading cause of premature death worldwide [1–3]. According to the World Health Organization, tobacco use causes more than 7 million deaths annually and reduces the life expectancy of smokers by 10 years compared with that of non-smokers [4]. Smoking cessation is strongly promoted as studies have shown that it reduces the risk of cardiovascular disease and death associated with smoking [5, 6].

The klotho gene, discovered in 1997, is closely linked to age-related diseases and aging progression [7]. High expression of the klotho gene in mammals is associated with extended lifespan, while low expression can accelerate aging [8]. The klotho protein exists as both a transmembrane protein and a secreted factor [9]. This study focuses specifically on serum α-klotho, the form of the α-klotho protein found in the bloodstream. Secreted klotho acts as a humoral factor that regulates ion channels, transporters, and reduces oxidative stress [10]. Serum α-klotho levels gradually decline with age after 40 [11]. Lower levels of serum α-klotho have been observed in individuals with cancer, cardiovascular disease, and diabetes, serving as a potential marker for increased mortality risk [12–15]. Thus, finding factors affecting serum α-klotho levels is crucial in studying human anti-aging problem. Previous studies have shown that smoking and cessation affect serum α-klotho levels [16–19].

The association between smoking, smoking cessation and serum α-klotho levels remains controversial. Japanese researchers found higher serum α-klotho levels in healthy male smokers compared with nonsmokers, while results in women were different and not statistically significant [16]. Turkish and American researchers observed lower serum klotho levels in smokers than nonsmokers in healthy men and preterm women, respectively [17, 18]. Furthermore, researchers found that smokers had lower serum α-klotho levels after quitting smoking than when they were smoking [19]. These studies show varying results on the effect of smoking and quitting smoking on serum α-klotho levels. All studies examined the connection between smoking, quitting smoking and serum α-klotho separately. This cross-sectional study aimed to examine the association between smoking, smoking cessation and serum α-klotho levels in the general population of the United States. Given the anti-aging properties of serum α-klotho protein, where overexpression promotes longevity while low expression is associated with premature aging symptoms, and taking into account the numerous risks associated with smoking and the benefits of smoking cessation. In this study, it was hypothesized that smoking may be negatively correlated with serum α-klotho levels, whereas smoking cessation may be positively correlated with serum α-klotho levels.

## Materials and methods

### Study design and population

This cross-sectional study used data from the 2013–2016 National Health and Nutrition Examination Survey (NHANES) conducted by the Centers for Disease Control and Prevention (CDC) [20]. The participants were selected using a multistage stratified probability process [21]. The NHANES study followed the principles of the Declaration of Helsinki. The National Center for Health Statistics collected information after obtaining approval from the Ethics Review Board. Written informed consent was obtained from all participants prior to their participation in the study [22]. The data collected in this study are publicly available on the NHANES website (http://www.cdc.gov/nchs/nhanes.htm).

This study focused on the adult population aged 40–79 years because serum α-klotho measurements were available only for this age group in the NHANES database. Participants with missing data regarding smoking status, serum α-klotho levels, or relevant covariates were also excluded. This study followed the Strengthening the Reporting of Observational Studies in Epidemiology reporting guidelines. The data applied in this study were anonymized and subjects’ personal information could not be identified.

### Measurement of serum α-klotho level

α-klotho concentration in fresh frozen serum samples was determined using a commercial enzyme-linked immunosorbent assay kit (IBL International, Japan) at a CDC-certified laboratory. The samples were stored at -80 °C. The reference range for α-klotho concentration was 285.8–1,638.6 pg/mL, with a mean of 698.0 pg/mL [11]. Quality assurance was achieved by calculating the average of two replicate analyses and providing a detailed description of the analytical method in a previous document [23]. The CDC performed all measurements as part of the ongoing NHANES.

### Exposure: Smoking

Participants‘ smoking status was determined using a two-question approach. The first question was "Have you smoked at least 100 cigarettes in your lifetime?" Those who responded negatively were classified as "never smokers." Those who responded positively proceeded to the second question, "Do you currently smoke cigarettes?" Participants who responded negatively were categorized as "ex-smokers," while those who responded "occasionally" or "daily" were classified as "current smokers" [4].

### Covariates

Based on previous studies, the following covariates were included in this study: age, sex, race/ethnicity, body mass index (BMI) [24, 25], alcohol status [26, 27], hypertension [28, 29], diabetes [12, 30], stroke [31, 32], liver disease [33, 34], CVD (coronary heart disease, angina, or congestive heart failure) [5, 13], CKD [35, 36], chronic obstructive pulmonary disease (COPD) [37, 38], and cancer [14, 39]. The race/ethnicity categories consisted of non-Hispanic White, non-Hispanic Black, Mexican American, and other races. BMI was categorized as < 30 and ≥ 30 kg/m2. Alcohol status was reported as yes (≥ 12 alcohol drinks/year) or no (< 12 alcohol drinks/year). Participants with an estimated glomerular filtration rate ≤ 60 mL/min/1.73 m2 or a urinary albumin/creatinine ratio ≥ 30 mg/g were classified as having CKD. Self-reported medical conditions included hypertension, diabetes, stroke, liver disease, CVD, COPD, and cancer, all of which were classified as yes or no.

### Statistical analysis

Categorical variables are expressed as proportions (%), while continuous variables are expressed as either the mean (standard deviation) or median (interquartile range), depending on the data distribution. Group differences were assessed using a one-way analysis of variance (for normally distributed data) and chi-square tests (for categorical variables).

Multivariate linear regression analysis models were used to examine the association between smoking, smoking cessation and serum α-klotho levels. Model 1 was unadjusted, whereas model 2 was adjusted for sociodemographic characteristics such as age, sex, and race/ethnicity. Model 3 was further adjusted for BMI and alcohol status. Model 4 was a fully adjusted model that included sociodemographic characteristics, BMI, alcohol status, diabetes, hypertension, stroke, CVD, liver disease, COPD, CKD, and cancer as covariates. Additionally, interaction and subgroup analyses were performed using linear regression models, considering age group (< 65 vs. ≥ 65 years), sex (male vs. female), race/ethnicity (non-Hispanic White vs. non-Hispanic Black vs. Mexican American vs. and other races). Given the minimal amount of missing data across all covariates (0–6.9%), a direct deletion approach was chosen to handle the missing data.

Sensitivity analyses were performed to ensure the robustness of our findings. Propensity score matching (PSM) was used to minimize potential confounders [40]. A 1:1 nearest neighbor matching algorithm was applied using a caliper width of 0.2. The variables listed above were selected as covariates to generate the propensity score. A standardized mean difference (SMD) was used to examine the degree of PSM. Less than 0.1 was considered an acceptable threshold.

Data analysis was performed with the statistical software packages R version 4.3.1 (http://www.R-project.org, The R Foundation) and Free Statistics version 1.8 (http://www.clinicalscientists.cn/freestatistics, Beijing, China). Statistical significance was determined using a two-tailed test with a threshold of *P* < 0.05.

## Results

### Characteristics of the participants

**Fig 1:**
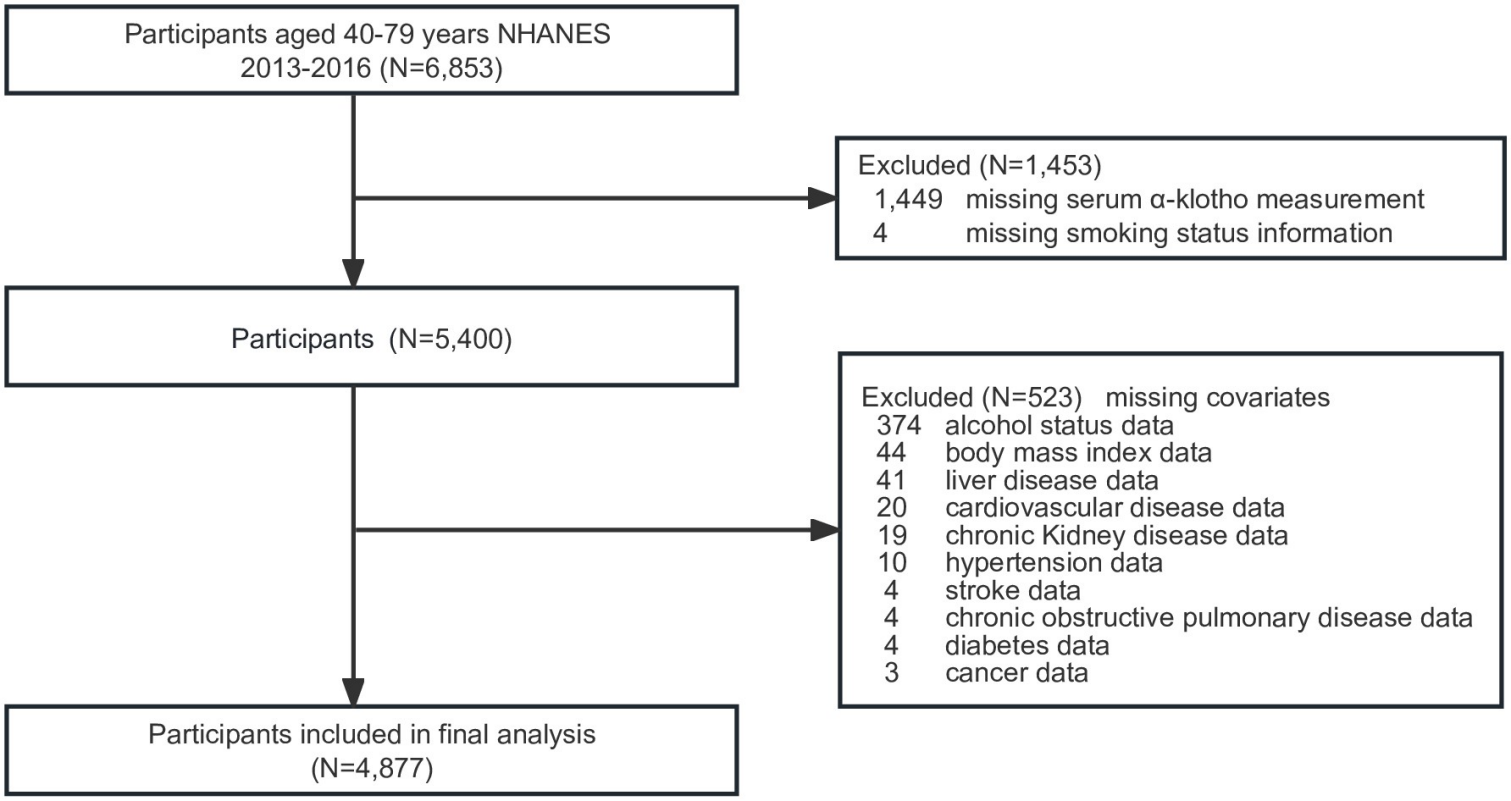
Flow diagram of the screening and enrollment of study participants.

From the initial sample of 6,853 participants aged 40–79 years, those with missing data were excluded: 1,449 for serum α-klotho data, 4 for smoking status information, and 523 for covariate data. A flowchart of participant enrollment is shown in Fig 1. As a result, a final sample of 4,877 was included in the study. This included 2,312 men (47.4%) and 894 current smokers (18.3%), and the mean age of the participants was 57.8±10.7 years. Current smokers had a younger age, decreased serum α-klotho levels, lower comorbid diabetes rates, and reduced BMI when compared with never-smokers. Conversely, current smokers had higher rates of male sex, non-Hispanic race, alcohol consumption, and comorbidities including hypertension, stroke, CVD, liver disease, COPD, CKD, and cancer than nonsmokers. Smoking cessation was less common in women than in men. Ex-smokers had higher serum α-klotho levels, BMI, rates of cancer, hypertension, diabetes, and CKD, and were older than current smokers. Moreover, quitters exhibited lower alcohol consumption, decreased prevalence of non-Hispanic Black ethnicity, and reduced incidence of stroke, cardiovascular disease, liver disease, and chronic obstructive pulmonary disease compared with current smokers (Table 1).

**Table 1:** Characteristics of participants in the NHANES 2013–2016 cycles.

Characteristic	Total	never-smokers	ex-smokers	current smokers	*P* value
	(n = 4877)	(n = 2600)	(n = 1383)	(n = 894)	
**Age, Mean(SD), yr**	57.8 (10.7)	56.7 (10.8)	61.1(10.3)	55.7 (9.8)	< 0.001
**Sex, n (%)**					< 0.001
**Male**	2312 (47.4)	1005 (38.7)	820 (59.3)	487 (54.5)	
**Female**	2565 (52.6)	1595 (61.3)	563 (40.7)	407 (45.5)	
**Race/ethnicity, n (%)**					< 0.001
**Non-Hispanic White**	1942 (39.8)	903 (34.7)	631 (45.6)	408 (45.6)	
**Non-Hispanic Black**	922 (18.9)	474 (18.2)	225 (16.3)	223 (24.9)	
**Mexican American**	806 (16.5)	484 (18.6)	215 (15.5)	107 (12)	
**Other Race**	1207 (24.7)	739 (28.4)	312 (22.6)	156 (17.4)	
**BMI, n (%)**					< 0.001
**<30kg/m2**	2787 (57.1)	1453 (55.9)	749 (54.2)	585 (65.4)	
**≥30kg/m2**	2090 (42.9)	1147 (44.1)	634 (45.8)	309 (34.6)	
**Alcohol status, n (%)**					< 0.001
**No**	1477 (30.3)	1138 (43.8)	213 (15.4)	126 (14.1)	
**Yes**	3400 (69.7)	1462 (56.2)	1170 (84.6)	768 (85.9)	
**Diabetes, n (%)**					< 0.001
**No**	3947 (80.9)	2124 (81.7)	1062 (76.8)	761 (85.1)	
**Yes**	930 (19.1)	476 (18.3)	321 (23.2)	133 (14.9)	
**Hypertension, n (%)**					< 0.001
**No**	3010 (61.7)	1677 (64.5)	793 (57.3)	540 (60.4)	
**Yes**	1867 (38.3)	923 (35.5)	590 (42.7)	354 (39.6)	
**Stroke, n (%)**					< 0.001
**No**	4670 (95.8)	2522 (97)	1313 (94.9)	835 (93.4)	
**Yes**	207 (4.2)	78 (3)	70 (5.1)	59 (6.6)	
**CVD, n (%)**					< 0.001
**No**	4431 (90.9)	2434 (93.6)	1221 (88.3)	776 (86.8)	
**Yes**	446 (9.1)	166 (6.4)	162 (11.7)	118 (13.2)	
**Liver disease, n (%)**					0.011
**No**	4730 (97.0)	2537 (97.6)	1338 (96.7)	855 (95.6)	
**Yes**	147 (3.0)	63 (2.4)	45 (3.3)	39 (4.4)	
**COPD, n (%)**					< 0.001
**No**	4661 (95.6)	2572 (98.9)	1301 (94.1)	788 (88.1)	
**Yes**	216 (4.4)	28 (1.1)	82 (5.9)	106 (11.9)	
**CKD, n (%)**					0.001
**No**	3702 (75.9)	2025 (77.9)	1007 (72.8)	670 (74.9)	
**Yes**	1175 (24.1)	575 (22.1)	376 (27.2)	224 (25.1)	
**Cancer, n (%)**					< 0.001
**No**	4291 (88.0)	2344 (90.2)	1154 (83.4)	793 (88.7)	
**Yes**	586 (12.0)	256 (9.8)	229 (16.6)	101 (11.3)	
**α-Klotho, Mean(SD), pg/mL**					< 0.001
	843.4 (304.6)	864.7 (289.1)	834.7 (334.4)	795.3 (294)	

Abbreviations: SD: standard deviation, BMI: body mass index, CVD: cardiovascular disease, CKD: chronic kidney disease, COPD: Chronic obstructive pulmonary disease.

### Multivariate linear regression analyses between smoking, smoking cessation and serum α-klotho levels

Tables 2 and 3 illustrate the results of the multivariate linear regression analyses examining the association between smoking, smoking cessation and serum α-klotho levels. In the unadjusted model, smoking and smoking cessation were inversely associated with serum α-klotho levels (β: -69.4, 95% confidence interval CI: -91.4 to -47.3 *p*<0.001; β: 39.4, 95% CI: 12.6 to 66.3 *p*<0.001). After adjusting for various confounders in model 4, including age, sex, race/ethnicity, BMI, alcohol status, hypertension, diabetes, stroke, liver disease, cancer, CVD, CKD, and COPD, these associations remained stable, with β values of -58.3 (95% CI: -82.0 to -34.6 *p*<0.001) and 52.3 (95% CI: 24.1 to 80.6 *p*<0.001), respectively.

**Table 2:** Association of smoking and serum α-klotho levels assessed by multiple linear regression models, NHANES 2013–2016, USA (N = 3,494).

Model	Participant, number.		Serum α-Klotho levels	*p-*value
	never-smokers^e^	current smokers^f^	*β* (95% CI)	
**Model1** ^a^	2600	894	-69.4(-91.4~-47.3)	<0.001
**Model2** ^b^	2600	894	-64.6(-87.0~-42.2)	<0.001
**Model3** ^c^	2600	894	-59.6(-82.6~-36.6)	<0.001
**Model4** ^d^	2600	894	-58.3(-82.0~-34.6)	<0.001

^a^Model1 was unadjusted.

^b^Model2 was adjusted for age, sex, and race/ethnicity.

^c^Model3 was adjusted for age, sex, race/ethnicity, ethnicity, body mass index, and alcohol status.

^d^Model4 was adjusted for age, sex, race/ethnicity, body mass index, alcohol status, diabetes, hypertension, stroke, cardiovascular disease (coronary heart disease, angina, and congestive heart failure), liver disease, chronic obstructive pulmonary disease, chronic kidney disease, and cancer.

^e^Never-smokers were defined as participants who reported smoking <100 cigarettes during their lifetime.

^f^Current smokers were defined as participants who smoked >100 cigarettes during their lifetime and who currently smoked.

Abbreviations: CI: confidence interval.

**Table 3:** Association of smoking cessation and serum α-klotho levels assessed by multiple linear regression models, NHANES 2013–2016, USA (N = 2,277).

Model	Participant, number.		Serum α-Klotho levels	*P* value
	ex-smokers^e^	current smokers^f^	*β* (95% CI)	
**Model1** ^a^	1383	894	39.4(12.6~66.3)	<0.001
**Model2** ^b^	1383	894	54.6(26.8~82.5)	<0.001
**Model3** ^c^	1383	894	55.5(27.4~83.5)	<0.001
**Model4** ^d^	1383	894	52.3(24.1~80.6)	<0.001

^a^Model1 was unadjusted.

^b^Model2 was adjusted for age, sex, and race/ethnicity.

^c^Model3 was adjusted for age, sex, race/ethnicity, ethnicity, body mass index, and alcohol status.

^d^Model4 was adjusted for age, sex, race/ethnicity, body mass index, alcohol status, diabetes, hypertension, stroke, cardiovascular disease (coronary heart disease, angina, and congestive heart failure), liver disease, chronic obstructive pulmonary disease, chronic kidney disease, and cancer.

^e^Ex-smokers were defined as participants who reported smoking >100 cigarettes during their lifetime and did not smoke currently.

^f^Current smokers were defined as participants who smoked >100 cigarettes during their lifetime and who currently smoked.

Abbreviations: CI: confidence interval.

### Subgroup and interaction analyses

S1 and S2 Figs show the association between smoking, cessation and serum α-klotho among age, sex, and race/ethnicity subgroups. The inverse correlation between smoking and serum α-klotho levels persisted across age, sex, and non-Hispanic subgroups. Nonetheless, this association was not present among Mexican Americans and other race. Furthermore, the *p* value for interaction between smoking and race on serum α-klotho levels was less than 0.001 (S1 Fig). The association between smoking cessation and serum α-klotho levels remained consistent in the subgroup of sex, participants younger than 65 years old, and non-Hispanic black individuals. However, correlations were not observed among those aged 65 years or older, as well as non-Hispanic white, Mexican American, and other race (S2 Fig).

### Sensitivity analyses

After applying PSM for the confounders of age, sex, race/ethnicity, BMI, alcohol status, hypertension, diabetes, stroke, liver disease, cancer, CVD, CKD, and COPD, associations between smoking, smoking cessation and serum α-klotho levels were still observed (β: -54.1; 95% CI: -81.5 to -26.7 *p*<0.001; β: 54.8; 95% CI: 24.2 to 85.4 *p*<0.001), as shown in Table 4.

**Table 4:** Sensitivity analyses.

Analysis	Participants,	Adjusted	*P* value
	number	*β* (95% CI)	
**Propensity score matching**			
**Never-smokers**^a^	802	1[Reference]	
**Current smokers**^b^	802	-54.1 (-81.5~-26.7)	<0.001
**Propensity score matching**			
**Current smokers**	761	1[Reference]	
**Ex-smokers**^c^	761	54.8 (24.2~85.4)	<0.001

The following variables were used to generate the model of Propensity Score Matching: age, sex, race and ethnicity, body mass index, alcohol status, diabetes, hypertension, stroke, cardiovascular disease (coronary heart disease, angina, and congestive heart failure), liver disease, chronic obstructive pulmonary disease, chronic kidney disease, and cancer.

^a^Never-smokers were defined as participants who reported smoking <100 cigarettes during their lifetime.

^b^Current smokers were defined as participants who smoked >100 cigarettes during their lifetime and who currently smoked.

^c^Ex-smokers were defined as participants who reported smoking >100 cigarettes during their lifetime, did not smoke currently.

Abbreviations: CI: confidence interval.

## Discussion

In this cross-sectional study, smoking was associated with reduced serum α-klotho levels, whereas smoking cessation was associated with elevated serum α-klotho levels. Our adjusted models and sensitivity analyses consistently revealed a robust association between smoking, smoking cessation and serum α-klotho levels.

In the present study, serum α-klotho levels were found to be lower in current smokers than in nonsmokers. Mustafa et al. found that serum α-klotho levels in healthy male smokers in Turkey were lower than those of nonsmokers, while Jennifer et al. reported similar findings in preterm female smokers in the United States [17, 18]. These findings are consistent with the results of the present study. Compared with previous studies, this study had a larger sample size, adjusted for more confounders, included both male and female participants, used the general population as the study population, and applied PSM for sensitivity analysis. The results of the present study are closer to real-world results.

Kaori et al. found higher serum α-klotho levels in healthy Japanese male smokers compared with nonsmokers. However, healthy female smokers showed slightly lower serum α-klotho levels than nonsmokers, without a statistically significant difference [41]. The inconsistency may be due to differences in the study population, sample size, confounding factors, and smoking intensity. Zoraida et al. found that serum α-klotho levels were higher in heavy smokers compared with light smokers [42]. researchers suggested that this increase might represent a compensatory response to smoking-induced stress, which subsequently decreases after smoking cessation.

In this study, serum α-klotho levels were observed to be higher in ex-smokers than in current smokers. However, a study of 28 Japanese smokers showed that after a 12-week smoking cessation intervention, post-quit levels were lower than baseline serum α-klotho levels [19], which is inconsistent with the results of this study. The discrepancy in the findings may be related to the different population, sample size, and smoking intensities. Compared with this Japanese study, the present study included non-smokers, ex-smokers, and current smokers to investigate the association between different smoking statuses and serum α-klotho levels. It was found that current smokers had lower serum α-klotho levels than non-smokers, while ex-smokers had higher levels than current smokers. These findings provide a complete chain of evidence for the smoking status on serum α-klotho levels.

In subgroup analyses, the *p*-value for the interaction between smoking and race on serum α-klotho levels was less than 0.001. Racial disparities may be linked to variations in tobacco brand preferences, smoking intensity, pharmacokinetics influenced by cytochrome P450 (CYP2A6) enzyme activity, and smoking habits [27]. The exact mechanism is unclear and needs to be further explored in subsequent studies.

Smoking is well known to stimulate the inflammatory cytokine IL-6 and promote inflammation [43]. IL-6 might induce fibroblast growth factor FGF23 expression through nuclear factor NF- κB activation [44]. FGF 23 is a potent negative regulator of α-klotho expression [45]. This partly explains the finding in the present study that current smokers have lower serum α-klotho levels than never smokers. The mechanism underlying the effect of smoking cessation on serum α-klotho levels remains unclear. We hypothesized that the reduction in inflammatory factors after smoking cessation leads to a downregulation of FGF 23 expression, which subsequently increases α-klotho expression. The specific mechanism needs to be further investigated in future studies.

## Limitations

There are some limitations to this study. First, the NHANES database only measures serum α-klotho levels in individuals aged 40–79 years, which limits the generalizability of our findings to other age groups. Second, there may be some residual confounders in this study, such as smog levels. However, We adjusted for possible confounders and minimized the influence of factors that could lead to outcome bias using PSM. Third, due to the lack of data on IL-6 in the NHANES database, its association with smoking and serum klotho could not be investigated in this study. Finally, we were unable to draw causal inferences due to the cross-sectional study design. Future prospective cohort studies are needed to investigate the causal relationship between smoking, smoking cessation and serum α-klotho levels.

## Conclusion

In summary, the present study found that serum α-klotho was lower in current smokers than in nonsmokers and higher in quitters than in current smokers. Our findings highlight the possibility that smoking and smoking cessation affect aging in opposite directions. Additional studies are required to validate these findings.

## Supporting information

S1 Fig Association between smoking and serum α-klotho levels by subgroup and interaction analyses, NHANES 2013–2016, USA (N = 3,494). Note: Each stratification was adjusted for age, sex, race and ethnicity, body mass index, alcohol status, hypertension, diabetes, stroke, liver disease, cancer, cardiovascular disease (coronary heart disease, angina, and congestive heart failure), chronic kidney disease(CKD), and chronic obstructive pulmonary disease except for the stratification factor itself. Circles represent the β coefficients, with horizontal lines indicating 95% confidence intervals (CIs). The diamonds represent the overall β coefficient, with the outer points of the diamonds indicating 95% CIs.
(TIF)

S2 Fig Association between smoking cessation and serum α-klotho levels by subgroup and interaction analyses, NHANES 2013–2016, USA (N = 2,277). Note: Each stratification was adjusted for age, sex, race and ethnicity, body mass index, alcohol status, hypertension, diabetes, stroke, liver disease, cancer, cardiovascular disease (coronary heart disease, angina, and congestive heart failure), chronic kidney disease(CKD), and chronic obstructive pulmonary disease except for the stratification factor itself. Circles represent the β coefficients, with horizontal lines indicating 95% confidence intervals (CIs). The diamonds represent the overall β coefficient, with the outer points of the diamonds indicating 95% CIs.
(TIF)
