# Supplementary figures and images for: Association between smoking, smoking cessation and serum α-klotho levels among American adults: National Health and Nutrition Examination Survey
Source: PLoS One. 2024 Mar 18;19(3):e0300562. doi: 10.1371/journal.pone.0300562 (PMC10947634; doi:10.1371/journal.pone.0300562)

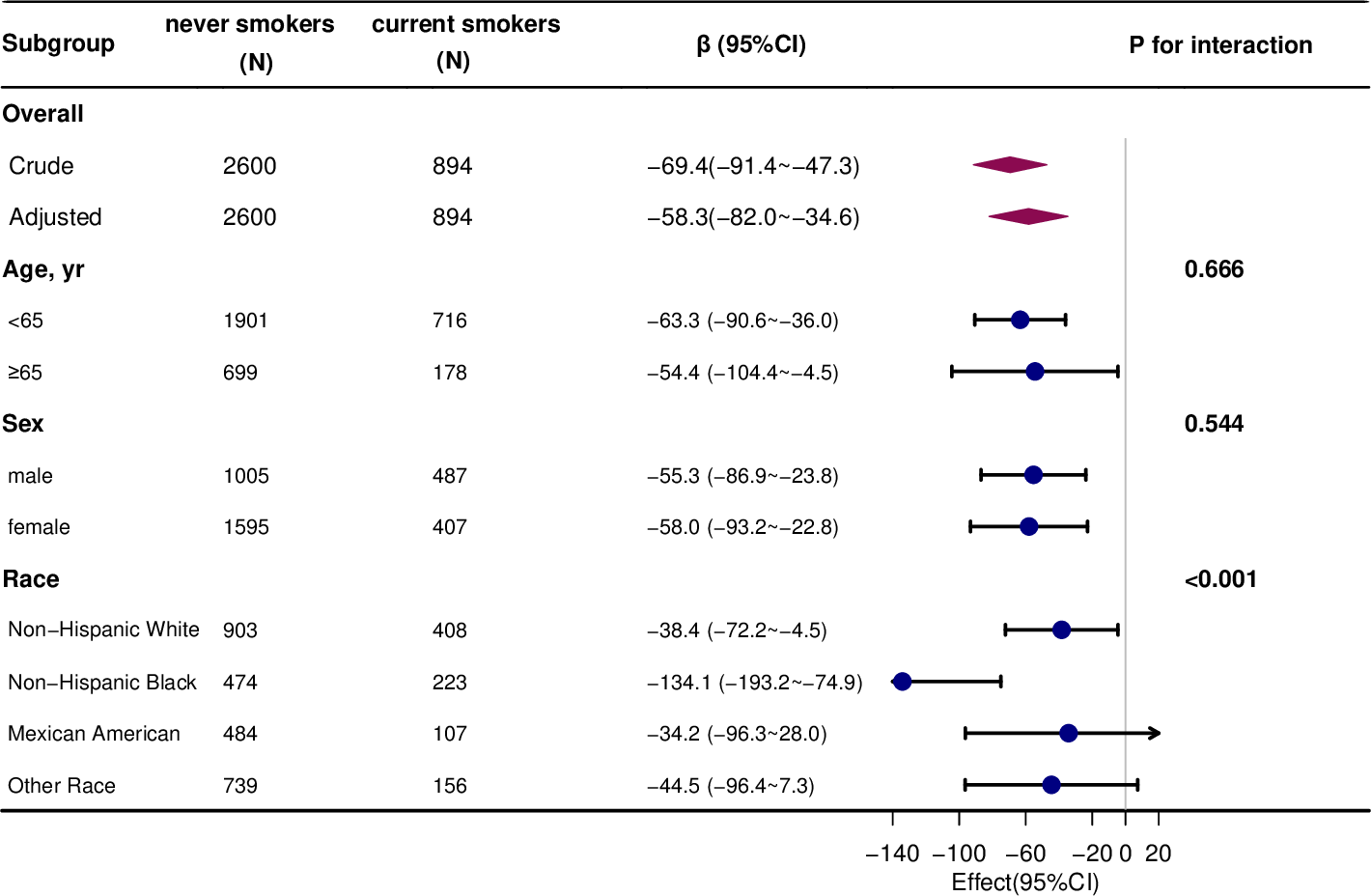

Supplement: S1 Fig — Note: Each stratification was adjusted for age, sex, race and ethnicity, body mass index, alcohol status, hypertension, diabetes, stroke, liver disease, cancer, cardiovascular disease (coronary heart disease, angina, and congestive heart failure), chronic kidney disease(CKD), and chronic obstructive pulmonary disease except for the stratification factor itself. Circles represent the β coefficients, with horizontal lines indicating 95% confidence intervals (CIs). The diamonds represent the overall β coefficient, with the outer points of the diamonds indicating 95% CIs. (TIF) [file pone.0300562.s001.tif]

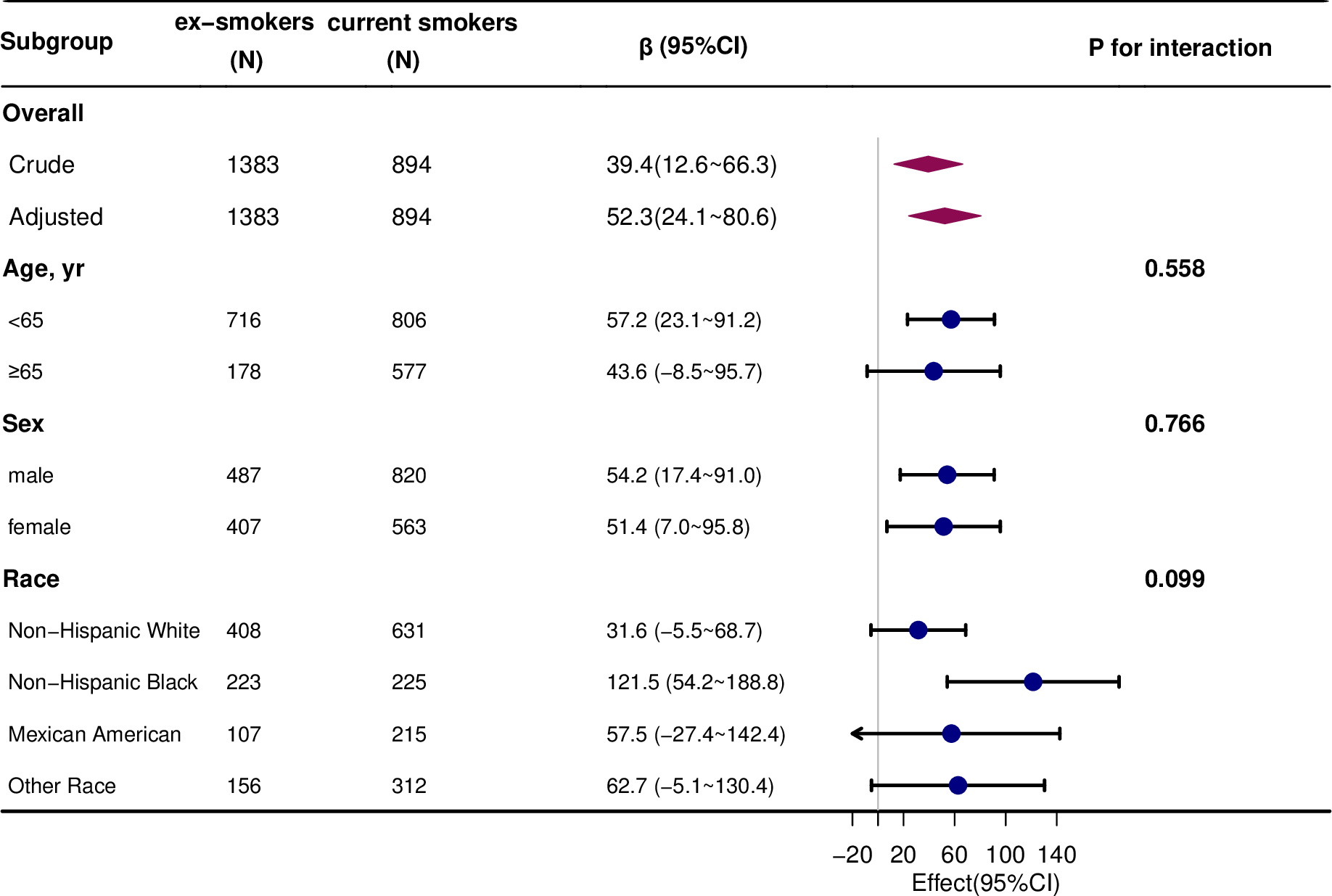

Supplement: S2 Fig — Note: Each stratification was adjusted for age, sex, race and ethnicity, body mass index, alcohol status, hypertension, diabetes, stroke, liver disease, cancer, cardiovascular disease (coronary heart disease, angina, and congestive heart failure), chronic kidney disease(CKD), and chronic obstructive pulmonary disease except for the stratification factor itself. Circles represent the β coefficients, with horizontal lines indicating 95% confidence intervals (CIs). The diamonds represent the overall β coefficient, with the outer points of the diamonds indicating 95% CIs. (TIF) [file pone.0300562.s002.tif]
